# Effects of ultrafiltration on systemic hemodynamics and microcirculatory perfusion in patients with end-stage kidney disease

**DOI:** 10.1186/cc9531

**Published:** 2011-03-11

**Authors:** E Klijn, M Van Genderen, M Betjes, J Bakker, J Van Bommel

**Affiliations:** 1 Erasmus MC University Medical Center, Rotterdam, the Netherlands

## Introduction

The relationship between systemic hemodynamic parameters and microcirculatory perfusion remains unclear. This is especially apparent in the concept of fluid responsiveness, where stroke volume (SV) can fluctuate strongly without being paralleled by changes in microcirculatory perfusion. Therefore, we hypothesized that large decreases in volume status due to ultrafiltration (UF) with intermittent hemodialysis in patients with end-stage kidney disease (ESKD) would decrease systemic hemodynamics but would not affect parameters of microcirculatory perfusion.

## Methods

Consecutive patients on chronic intermittent hemodialysis for ESKD were eligible for our study. SV and heart rate were measured continuously and non-invasively using NICOM, a technique based on chest bioreactance. Blood pressure was measured intermittently with a sphygmomanometer. Peripheral and microcirculatory perfusion were measured intermittently with sidestream dark-field (SDF) imaging (sublingual area), and continuously with forearm-to-finger temperature gradient (Tskin-diff) and photopletysmography (PPG) (finger). All parameters were assessed before (baseline) and after 4 hours at the end of UF.

## Results

Data are presented as median (IQR). Twenty-one patients (13 males, median age 59 (51 to 66) years) were included in our study. A median volume of 2,200 (1,850 to 2,850) ml was removed. SV and mean arterial pressure decreased during UF from 75 (58 to 84) ml to 51 (37 to 67) ml (*P *< 0.01) and from 102 (88 to 109) mmHg to 85 (75 to 95) mmHg (*P *< 0.001), respectively, while heart rate did not change. At baseline all parameters of peripheral and microcirculatory perfusion were undisturbed. During UF, Tskin-diff and the PPG of the finger did not change. Sublingual microvascular flow index and vessel density measured with SDF slightly decreased from 3.0 (3.0 to 3.0) to 2.8 (2.7 to 2.9) (*P *< 0.001) and from 10.6 (9.9 to 11.1) n/mm to 9.9 (9.2 to 10.5) n/mm (*P *< 0.05), respectively.

## Conclusions

UF leads to a significant and uniform decrease in volume status in patients with ESKD but surprisingly this was not associated with large decreases in peripheral and microcirculatory perfusion. Therefore caution is warranted when interpreting systemic hemodynamic parameters in terms of hypovolemia and hypoperfusion when peripheral perfusion is not evidently impaired.

